# Development of capillary dysfunction in Alzheimer’s disease

**DOI:** 10.3389/fnagi.2024.1458455

**Published:** 2024-08-29

**Authors:** S. Vorobev, S. Yanishevskiy, S. Efimtsev, A. Sokolov, V. Dyachuk

**Affiliations:** Laboratory of Neurogenesis and Neurodevelopmental Disorders, World-Class Research Center for Personalized Medicine, Almazov Center, Saint-Petersburg, Russia

**Keywords:** neurodegeneration, capillary dysfunction, mitochondrial dysfunction, oxidative stress, β-secretase pathway, BBB function, glymphatic system, amyloid β buildup

## Abstract

Alzheimer’s disease (AD) is currently considered the major cause of cognitive impairment in older adults. This explains the close attention to the issue of AD research. The pathomorphological basis of the disease is a neurodegenerative process, the early stages of which are formed in the hippocampus and the morphofunctionally deep parts of the temporal lobes of the brain closely related to it. Several hypotheses have been advanced concerning the causes of neurodegeneration: the amyloid hypothesis, the calcium homeostasis impairment hypothesis, the inflammatory hypothesis, and the prion hypothesis. However, these hypotheses cannot explain the early stages of the pathogenesis of neurodegenerative diseases, in particular Alzheimer’s disease. This health problem requires further comprehensive study of available data, as well as additional investigations to determine the nature of such a process. In this review, the data on microcirculatory disorders in the capillaries of the hippocampus and mediobasal structures of the temporal lobes of the brain, which may be an initiating factor that triggers neurodegenerative events, are analyzed.

## Introduction

Numerous studies available in the literature consider the pathogenesis of neurodegeneration in general and Alzheimer’s disease (AD) in particular. The amyloid hypothesis has recently been a key one. According to it, the development of the disease is caused by the aggregation of pathologic amyloid β protein into senile plaques. In health, the amyloid β precursor protein (APP) normally undergoes sequential posttranslational proteolysis by two proteases. Due to the initial action of α-secretase, a soluble fragment, sAPP-α, and a membrane-bound C-terminal region are observed to form. The exposure of the latter to γ-secretase leads to the formation of the p3 fragment. Both substances are soluble and are involved in maintaining a number of crucial reactions such as neuroplasticity, synaptic transmission, signaling function, and neurogenesis (Ermilov and Nesterova, 2016; Nalivaeva, 2022). In pathology, the β-secretase pathway of APP proteolysis becomes dominant instead of the α-secretase pathway. Formation of two proteins is observed under its effect. The first one is the peptide sAPP-β, which is soluble but cannot fully perform its physiological functions due to a slightly different configuration with a lower number of amino acid residues compared to sAPP-α. The second is the membrane-bound C-terminal fragment C99 consisting of 99 amino acid residues. Subsequently, the amyloid β protein is formed from it when under the effect of γ-secretase. It is able to spontaneously aggregate, passing through several intermediate forms. These forms include oligomerization of the protein into fragments containing from two to six peptides, protofibrils, and fibrils that form amyloid plaques. Recent studies show that soluble forms of the amyloid β protein exhibit a significantly greater neurotoxic effect compared to the protein’s aggregated form (Litvinenko et al., 2019; Chumakov et al., 2020).

Another hypothesis of AD development considers the impairment of calcium homeostasis. Ca^++^ plays a significant role in the organization of intracellular signaling, being the second most important messenger involved in the regulation of neuronal activity. Its excess accumulation in the cell leads to the activation of intracellular enzyme systems (proteases, endonucleases, etc.). Simultaneously, a cascade of reactions of slow excitotoxicity and the induction of oxidative stress occur. These factors combined result in degeneration of cellular structures (Popugaeva et al., 2018; Bezprozvanny, 2022). The inflammatory hypothesis of AD formation and the somewhat related glial hypothesis have become of great importance. As a significant number of studies have shown, during the development of neurodegeneration in brain tissue, cerebrospinal fluid, and blood serum, there is a significant increase in the amount of proinflammatory cytokines and other inflammatory process markers. The activation of microglia, which is the main immune-presenting factor of the brain, plays a particular role. Astrocytes, which perform polymodal functions within the central nervous system, are also of crucial importance. Their activation along with immune and inflammatory responses can lead to dysfunction of the glymphatic system, lower efficiency of synaptic transmission, and also additional synthesis of amyloid β (Vorobev et al., 2020; Kushnireva et al., 2019). The prion hypothesis is rather interesting. It is based on the data obtained through several studies on the prion-like properties of the amyloid β protein. In particular, improper arrangement of the molecule into the tertiary structure is observed, resulting in alteration of protein’s properties. Moreover, it was found that upon contact with a normal protein the spatial arrangement of this molecule changes and it leads to the spread of pathology (Tatarnikova et al., 2015). There are also other hypotheses. Although being undoubtedly important in terms of revealing certain aspects of AD development, they all have one disadvantage. The changes described in them require a trigger factor that would be an initial cause of the considered events. Nevertheless, these hypotheses cannot explain the early stages of AD pathogenesis. The current state of the problem necessitates a comprehensive analysis of available data and additional studies that could help identify the nature of such a process.

A systematic overview of the studies carried out in this field to date has demonstrated that one of the factors initiating neurodegeneration may be a local microcirculatory impairment occurring in capillaries of the hippocampus and mediobasal structures of the temporal lobes of the brain. Thus, a morphological study of hippocampal tissue samples from middle-aged individuals (up to 60 years old) revealed changes indicating ischemic cell damage along with impaired blood circulation in the vessels of the microcirculatory bed (Gorelik, 2014). Thus, involutional changes in capillaries in old age were significantly higher in the regions of the archicortex, including the hippocampus, than in the neocortex (Pigolkin and Zolotenkova, 2014). Also, several experimental studies revealed a decrease in the number, density, and length of capillaries in the hippocampus and several cortical areas in aged rats. These data are generally consistent with the results of clinical observations that reported a decrease in these parameters in groups of individuals older than 75 years (Brown and Thore, 2011). Moreover, a 30% decrease in capillary density was found in the hippocampus and cerebral cortex compared to healthy animals in a study based on an experimental model of AD (Paris et al., 2004). In a study of brain tissue samples from patients who suffered from AD during their lifetime, a decrease in the microcirculatory bed density was also found compared to the age norm (Brown et al., 2007). It should be noted that the heterogeneity of capillary blood flow found in the temporoparietal regions, cingulate gyrus, and precuneus correlated negatively with the results of the mini-mental state examination (MMSE) in patients with probable and possible Alzheimer’s disease (Østergaard et al., 2015). Another study showed a decrease in the intercapillary distance in hippocampal and cortical tissue samples from patients with Alzheimer’s disease compared to cognitively intact individuals of a similar age. The authors characterize such changes in terms of changes in angiogenesis and vascular remodeling aimed to eliminate the observed capillary dysfunction (Kirabali et al., 2020).

**Figure 1:**
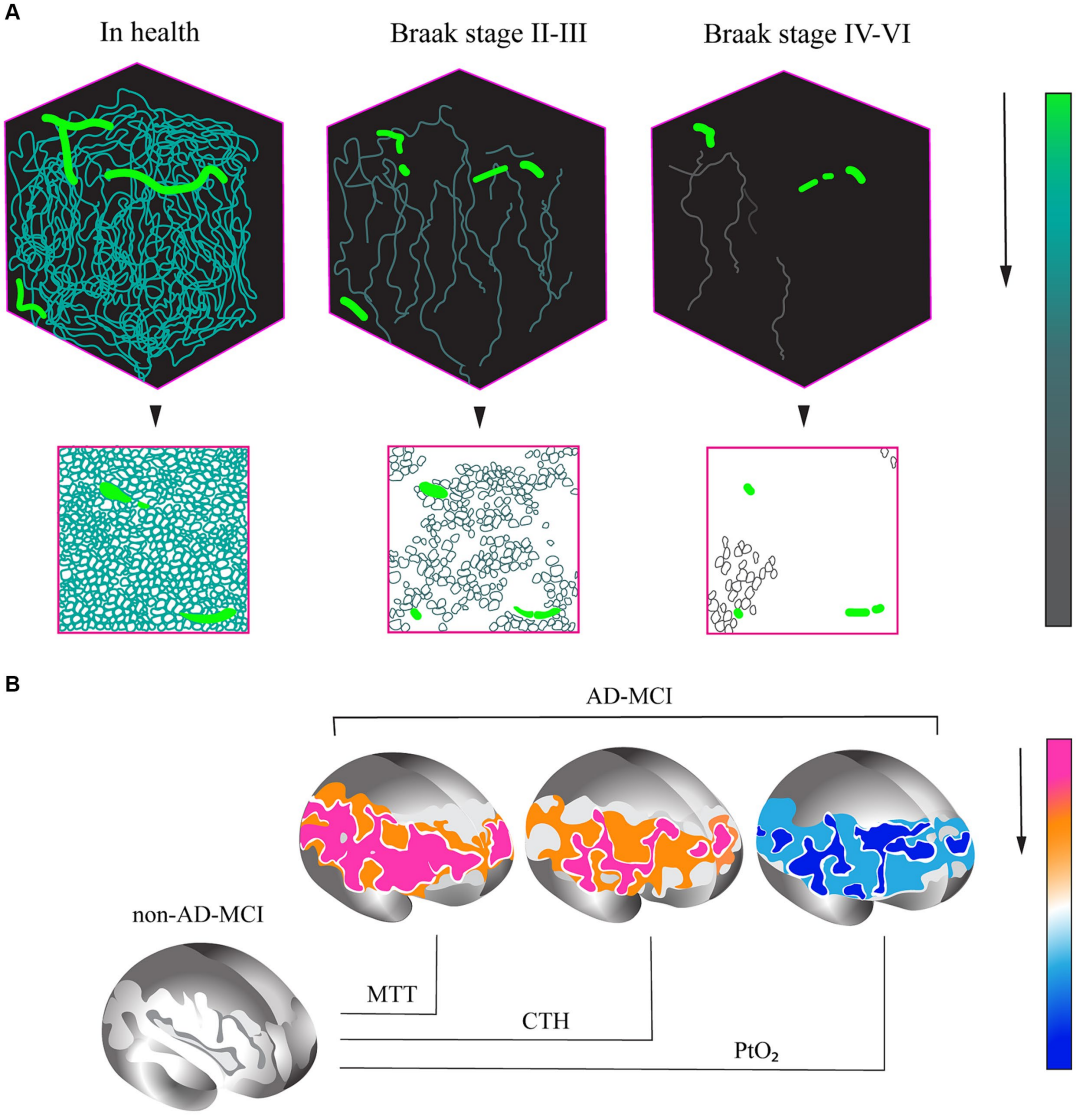
**(A)** Variations in capillary density in tissue samples of CA1 hippocampal region in AD patients and healthy control individuals without cognitive impairment. **(A)** Evaluation of capillary density based on immunofluorescence analysis of PECAM (platelet/endothelial cell adhesion molecule) or CD31 endothelial cell marker accumulation. **(B)** Percentage variation in capillary density at different stages of AD. (1) In health. (2) AD, Braak stages II–III. (3) AD, Braak stages IV–VI (base on research from Millien et al., 2022). **(B)** Changes in capillary perfusion determined via spin echo-based perfusion weighted MRI. A decrease in a number of parameters in patients with the prodromal AD (AD-MCI, MCI of the Alzheimer type) relative to patients with the non-Alzheimer type (non-AD-MCI) is shown. MTT, mean transit time; CTH, capillary transit time heterogeneity; P_t_O_2_, tissue oxygen tension (cited from Madsen et al., 2022).

This explanation can be supported by the results of another study, which revealed a reduced capillary density in tissue samples from the CA1 hippocampal region of patients who suffered from Alzheimer’s disease in their lifetime at Braak stages II–III, compared to samples obtained from patients who did not suffer from cognitive impairment. Moreover, the capillary density in the hippocampus increased at Braak stages IV–VI (Figure 1) (Millien et al., 2022).

An overview of 37 studies that assessed the capillary bed in aging and AD has also allowed the identification of several general patterns typical of these conditions. In particular, a number of specific changes indicating the development of capillary dysfunction were detected in older adults, as well as in patients with clinical manifestation of AD and in its model in laboratory animals. This phenomenon is manifested as a decrease in capillary density, increase in intercapillary distance, and decrease in vessel length (Brown and Thore, 2011).

The available pathomorphology data indicating the active role of capillary dysfunction in the AD development has contributed to the studies aimed at confirming this hypothesis *in vivo*. In view of the specifics of such studies on patients *in vivo*, the methods for the assessment of capillary blood flow based on magnetic resonance are promising. Significant efforts have been made in this regard. For instance, 32 patients with diagnosed AD, who had the MMSE score of more than 18, were examined in one of the protocols. Pittsburgh compound B positron emission tomography (PiB-PET) was obligatory performed on the patients. Magnetic resonance imaging (MRI) was performed by a 3 T Magnetom Tim Trio system, and perfusion-weighted MRI was conducted by both gradient-echo (GE) and spin-echo (SE) echo-planar imaging. Cortical thickness was measured on T1w images and compared to a standard cortical surface in Montreal Neurological Institute (MNI) space. Postprocessing analysis was carried out using the SPM8 statistical parametric mapping software package. Cerebral blood flow, blood volume in the microvascular bed, mean blood transit time, capillary transit time heterogeneity, and tissue oxygen tension were assessed using a special methodology. It was found that initially worse results of the cognitive assessment and lower parameters of the cortical thickness correlated with decreased cerebral blood flow in general and worse capillary blood flow in particular. These were also detected against the backdrop of higher blood flow velocity, heterogeneity of blood transit time through the microcirculatory bed, and low oxygen tension parameters. The reassessment performed 6 months later demonstrated a significant relationship of the degree of higher cortical function impairment with the increase in the relative heterogeneity of capillary transit times (Nielsen et al., 2017).

**Figure 2:**
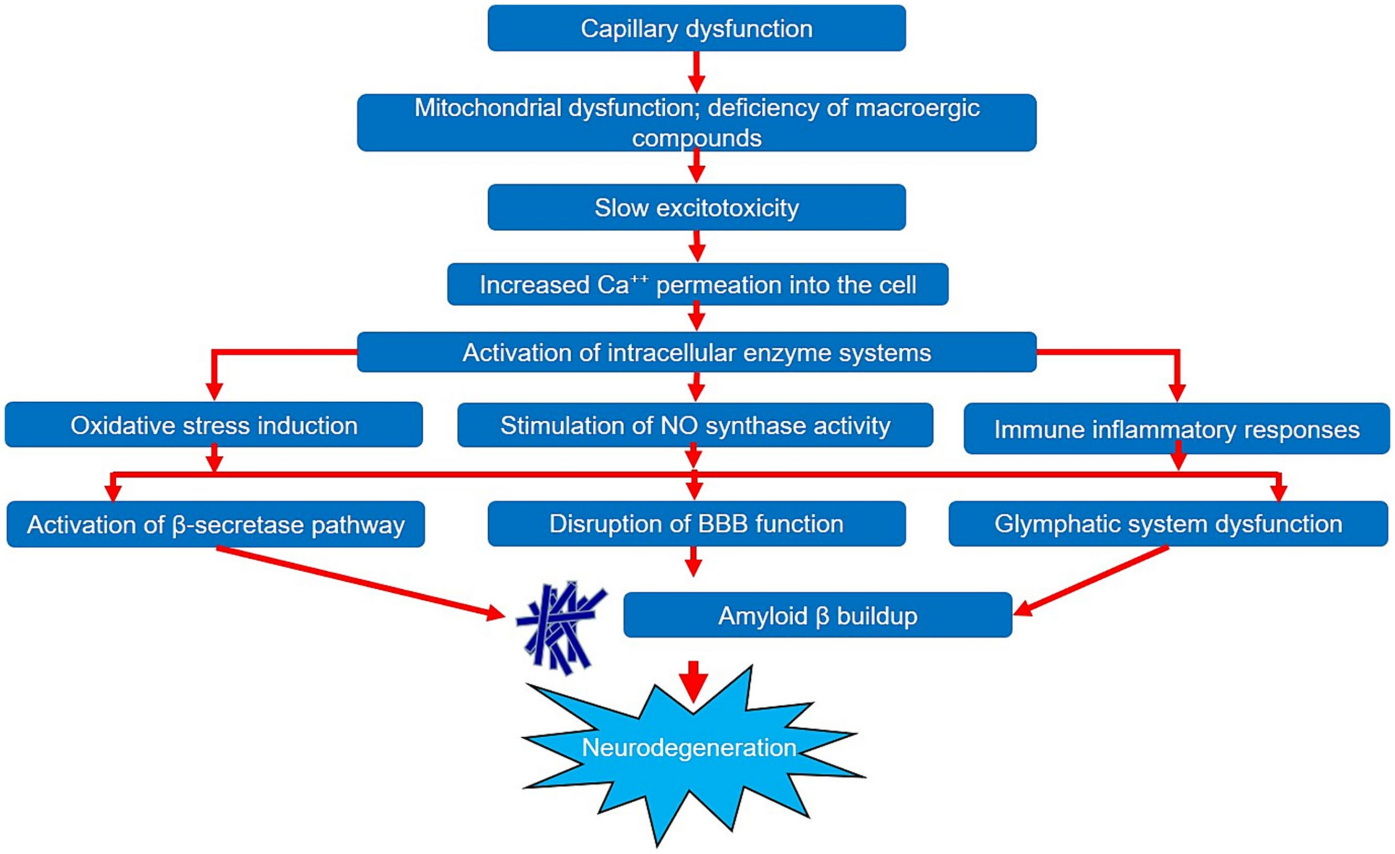
General diagram of impact of capillary dysfunction on neurodegeneration development.

In another study, a comparative assessment of changes in capillary blood flow in groups of patients with moderate cognitive impairment (MCI) of Alzheimer and non-Alzheimer types was conducted. As a result, the patients with prodromal AD (MCI) showed a significant increase in the capillary blood flow and transit time heterogeneity and also a decrease in tissue oxygen tension in the brain during 2 years of observation (Figure 2) (Madsen et al., 2022).

Arterial spin labeling (ASL) MRI, which is a contrast-free MR perfusion technique, revealed a decrease in blood flow in the posterior cingulated, lingual gyri, and also in the hippocampus in patients with amnestic MCI and mild dementia of the Alzheimer type compared to the group of individuals without cognitive impairment (Soman et al., 2021). Another study was aimed at investigating the correlation between microcirculation dysfunction and amyloid β aggregation in patients with prodromal AD. To this end, dynamic susceptibility contrast MRI and PET with Pittsburgh compound B were performed. There was a significant relationship between the decreased microvascular blood flow and its increased heterogeneity on one side and amyloid β deposition in temporal and frontal lobes on the other (Madsen et al., 2023). The changes identified in the study on the relationship between microcirculatory dysfunction and tau deposition were not so unambiguous. In particular, spin-echo and gradient echo dynamic susceptibility contrast magnetic resonance imaging (MRI) with intravenous injection of gadolinium-based contrast agent and using flortaucipir PET, which is a tau ligand, were performed on patients with MCI or mild dementia in AD. The results showed a reduced global cortical cerebral blood flow, variations in microvascular cerebral blood flow, an increase in capillary transit heterogeneity, as well as changes in the oxygen extraction fraction level in the cortex (Bryant et al., 2021).

The presented results of pathomorphological and clinical laboratory studies suggest the probable development of capillary dysfunction in early stages of Alzheimer’s disease. The formation of capillary dysfunction contributes to chronic ischemic damage of the brain matter, which triggers a cascade of biochemical and pathogenic reactions, thus, causing neurodegeneration. In health, the cerebral capillary network has a high density and quite a complex structure with various loops and branches, which allows an optimal trophic support of cellular elements of the brain (Erdener and Dalkara, 2019). With aging, the efficiency of oxygen delivery to brain tissue decreases in addition to the combined effect of vascular risk factors and changes in the vascular bed. However, in young and middle age, these processes are compensated by sufficient capillary force. In older age, against the backdrop of progressive macrovascular changes, a reduction in the relative density of the capillary bed and a decrease in the number of branches can be observed in some cases, which leads to hypoxic–ischemic damage to neurovascular unit structures (Moeini et al., 2018). One of the consequences of cerebral tissue ischemia is the development of mitochondrial dysfunction with a deficiency of macroergic compounds. Along with this, a cascade of reactions known as metabolic (slow) excitotoxicity is observed. It is based on the removal of the magnesium block of NMDA-type glutamate receptors in the context of disruption of ATP-dependent systems. It leads, in turn, to the increased Ca^++^ ion permeation into the cell with subsequent activation of intracellular enzyme systems, degeneration of neurons, and release of endogenous glutamate (Ong et al., 2013).

Against this backdrop, the induction of oxidative stress is also observed, accompanied by the damage of biological molecules, the release of arachidonic acid, the formation of thromboxanes, prostaglandins, and leukotrienes, and the excessive synthesis of free radicals. Moreover, the increased intracellular accumulation of Ca^++^ ions leads to the stimulation of NO synthase activity with the production of nitric oxide, which contributes to the disruption of oxidative phosphorylation in mitochondria with the release of reactive oxygen species. Both of these processes activate lipid peroxidation and cause additional cell damage. In addition, oxidative stress triggers the apoptosis process by interfering with the modulation of several signaling pathways such as ERK1/2, Nrf2, etc. (Nikolenko et al., 2022; Dhapola et al., 2024).

Another important consequence of slow excitotoxicity is the development of chronic immune and inflammatory responses, described under the term “Inflammaging,” which is accompanied by the accumulation of several proinflammatory cytokines such as IL-1, IL-6, TNF-α, C-reactive protein, and leukocyte elastase in brain matter and blood serum (Cornejo and von Bernhardi, 2016; McGuire, 2019).

These processes, exhibiting mutual potentiation, increase the likelihood of neurodegeneration due to their ability to increase the build-up of pathologic amyloid β protein in the brain. This effect can be exerted in three different ways.

The first way consists in the increase in the β-secretase pathway of proteolysis of the APP precursor protein. Studies have shown both a significant build-up of β-secretase (BACE1) amount and an increase in its gene expression in the brain tissues of patients with Alzheimer’s disease (Tan and Evin, 2012; Kocki et al., 2015). Currently, several variants of such an effect have been described. The regulatory protein HIFα, the hypoxia-inducible factor alpha, plays an important role in this process. With normal oxygen content, this protein undergoes degradation. Its build-up is observed in the case of hypoxia development (Novikov and Levchenkova, 2013). The BACE1 gene contains a functional region in its promoter that responds to hypoxia. By having an effect on it, HIFα contributes to the BACE1 gene expression with the subsequent increase in the amyloidogenic pathway of APP processing (Sun et al., 2012). Another mechanism is explained by the depletion of the transport proteins GGA1 and GGA3 capable of binding to ADP-ribosylation factor in ischemia. GGA1 has been shown to promote the BACE1 protein transport from endosomes to the Golgi apparatus for subsequent elimination. GGA3 is responsible for transporting BACE1 into lysosomes for further degradation. GGA3 is responsible for transporting BACE1 into lysosomes for further degradation. It was found that the observed decrease in the contents of both GGA1 and GGA3 in AD correlates with the increase in BACE1 and the increased postprocessing proteolysis of the protein APP through the amyloidogenic pathway. Overexpression, in particular, of the GGA1 protein is, *vise versa*, accompanied by APP cleavage with the formation of non-amyloid soluble fragments (Tan and Evin, 2012; Kosicek et al., 2014). Another factor that can trigger the relative activity of the β-secretase pathway is the disruption of the ubiquitin-mediated system. In health, its function consists in proteolysis and degradation of damaged and malfunctioning proteins with pathological spatial structures. The proteasome itself is a multiprotein complex with proteolytic activity and the ability to cleave target proteins into individual peptides. The binding of this protein to ubiquitin is essential for the initiation of such an action (Kudriaeva and Belogurov, 2019). Along with the cerebral tissue ischemia, there is an excessive accumulation of damaged protein molecules binding to ubiquitin. This leads to overstimulation of proteasomes and a gradual increase in ubiquitinated aggregates containing pathological proteins. The reduced proteasomal activity is further provoked by a decrease in the amount of ATP formed in brain tissue hypoxia. These changes result in the buildup of amyloid β protein (Riederer et al., 2011; Caldeira et al., 2014).

The second way of the amyloid β protein buildup may be related to the dysfunction of the blood-brain barrier (BBB). A number of changes are known to accumulate in ischemia such as endothelial dystrophy, irregular thickness of basement membranes, swelling of the surrounding astrocytic endfeet, and others, which lead to the disruption of the BBB function (Østergaard et al., 2013). Impaired functioning of transporters is observed against this backdrop, which affects the quality of amyloid β elimination into blood serum. Pericyte loss, which is observed under reduced capillary blood flow, is also of great importance. Pericytes play a significant role in the regulation of BBB permeability due to their ability to be involved in the formation of tight contacts and stabilize endothelial cells. Their degradation leads to a decrease in the amyloid β clearance. In addition, amyloid β oligomers alone can damage pericytes and reduce the efficiency of capillary blood flow. Thus, a vicious circle is formed, in which microcirculation disturbance and amyloid β aggregation mutually increase (Madsen et al., 2023).

The third way of increasing the amyloid β content in brain tissues is associated to glymphatic system dysfunction. One of its most important components are astrocytes, which form a continuous sheath around vessels in the brain parenchyma with their endfeet. Aquaporin-4 protein was found to be one of the main components of these endfeet, covering most of their surface (Nagelhus and Ottersen, 2013). Morphologically, these are a transmembrane protein complex consisting of four homologous subunits, each having a water channel inside. The elimination of various substances, including amyloid β, into paravenous spaces occurs through this channel. Its active role in this process was confirmed experimentally through assessing the movement of the amyloid β 1–40 protein in laboratory animals. Besides, the aquaporin-4 gene knockout was found to cause a 55% decrease in the elimination of amyloid β from the brain matter (Iliff et al., 2012). It was shown that, with aging, the glymphatic system’s efficiency sharply deteriorates, which leads to a decrease in its ability to eliminate substances. Several possible mechanisms for this phenomenon are under discussion. With age, against the background of hypoxic changes, the thickening of endothelium and an increase in its stiffness are observed, which hinders arteriole pulsation and reduces the efficiency of cerebrospinal fluid flow through the paravascular spaces (Kress et al., 2014). In addition, the developing “Inflammaging” process promotes the transition of astrocytes to an activated state, which negatively affects the glymphatic system functions (Guzman-Martinez et al., 2019). It was also found that during AD development, there is a significant decrease in the amount of aquaporin-4 in astrocytic endfeet, with a simultaneous increase in those parts of the cell that are not in direct contact with microcirculatory bed vessels. Interestingly, these changes are registered as early as at the prodromal stage of the disease and correlate with the values of such tests as the MMSE and clinical rating scale. This may suggest a primary impairment of aquaporin-4 polarization relative to the impaired clearance of amyloid β protein (Simon et al., 2022). However, this matter remains a subject of debate.

Thus, we can state that a large amount of data has been collected to date on the significant impact of capillary dysfunction during the onset of the neurodegenerative process in AD. Deterioration of blood supply at the level of microcirculation triggers a cascade of pathobiochemical and pathophysiological reactions, leading to the development of hypoxia and chronic ischemic damage to brain tissues. These reactions are quite diverse and can have a pathological effect on various components and mutually potentiate each other. The general pattern of the impact of capillary dysfunction on the neurodegeneration development is shown in Figure 2. It should also be noted that a number of studies provide evidence of the primacy of microcirculatory changes relative to such significant components of AD pathogenesis as the accumulation and aggregation of the amyloid β protein. However, additional studies are required to confirm this statement, which will help to reliably determine the role of capillary dysfunction in the AD pathogenesis. Studying the microcirculation features will certainly extend our knowledge of the pathogenesis of both AD and neurodegeneration in general and will ultimately contribute to the improvement of patient management strategies in this cohort.

## Conclusion

Currently, a large amount of data has been accumulated that goes beyond the amyloid theory of the pathogenesis of Alzheimer’s disease. Some of them indicate a possible active role in this process of capillary insufficiency. The available information indicates the presence of similar changes in the microcirculatory bed during the pathomorphological study of brain tissues in laboratory animals with simulated Alzheimer’s disease and during histological examination of samples obtained from samples of the hippocampus and other departments of patients who suffered from this disease during their lifetime. Similar changes were recorded in the postprocessing analysis of data obtained using special MR methods. Clarifying the effect of capillary insufficiency on the formation of Alzheimer’s disease will contribute to improving our understanding of its pathogenesis.

## Author contributions

SV: Conceptualization, Data curation, Investigation, Methodology, Validation, Writing – original draft, Writing – review & editing. SY: Conceptualization, Data curation, Investigation, Methodology, Validation, Writing – original draft, Writing – review & editing. SE: Data curation, Formal analysis, Investigation, Methodology, Writing – review & editing. AS: Data curation, Formal analysis, Methodology, Writing – review & editing. VD: Conceptualization, Formal analysis, Funding acquisition, Project administration, Supervision, Writing – original draft, Writing – review & editing.

## References

[ref1] Bezprozvanny I. (2022). Alzheimer’s disease—where do we go from here? Biochem. Biophys. Res. Commun. 633, 72–76. doi: 10.1016/j.bbrc.2022.08.075 36344168

[ref2] Brown W. R. Moody D. M. Thore C. R. Challa V. R. Anstrom J. A. (2007). Vascular dementia in leukoaraiosis may be a consequence of capillary loss not only in the lesions, but in normal-appearing white matter and cortex as well. J. Neurol. Sci. 257, 62–66. doi: 10.1016/j.jns.2007.01.015, PMID: 17320909 PMC1989116

[ref3] Brown W. R. Thore C. R. (2011). Review: cerebral microvascular pathology in ageing and neurodegeneration. Neuropathol. Appl. Neurobiol. 37, 56–74. doi: 10.1111/j.1365-2990.2010.01139.x, PMID: 20946471 PMC3020267

[ref4] Bryant A. G. Manhard M. K. Salat D. H. Rosen B. R. Hyman B. T. Johnson K. A.. (2021). Heterogeneity of tau deposition and microvascular involvement in MCI and AD. Curr. Alzheimer Res. 18, 711–720. doi: 10.2174/1567205018666211126113904 34825871 PMC8822690

[ref5] Caldeira M. V. Salazar I. L. Curcio M. Canzoniero L. M. Duarte C. B. (2014). Role of the ubiquitin-proteasome system in brain ischemia: friend or foe? Prog. Neurobiol. 112, 50–69. doi: 10.1016/j.pneurobio.2013.10.003 24157661

[ref6] Chumakov N. M. Luzina E. A. Demenkova I. S. Spirina M. A. Shamrova E. A. Makarova Y. A.. (2020). Modern concept of pathogenesis of Alzheimer’s disease. Mod. Probl. Sci. Educ. 2:164. doi: 10.17513/spno.29647

[ref7] Cornejo F. von Bernhardi R. (2016). Age-dependent changes in the activation and regulation of microglia. Adv. Exp. Med. Biol. 949, 205–226. doi: 10.1007/978-3-319-40764-7_10, PMID: 27714691

[ref8] Dhapola R. Beura S. K. Sharma P. Singh S. K. HariKrishnaReddy D. (2024). Oxidative stress in Alzheimer’s disease: current knowledge of signaling pathways and therapeutics. Mol. Biol. Rep. 51:48. doi: 10.1007/s11033-023-09021-z, PMID: 38165499

[ref9] Erdener Ş. E. Dalkara T. (2019). Small vessels are a big problem in neurodegeneration and neuroprotection. Front. Neurol. 10:889. doi: 10.3389/fneur.2019.00889, PMID: 31474933 PMC6707104

[ref10] Ermilov V. V. Nesterova A. A. (2016). β-amyloidopathy in the pathogenesis of age-related macular degeneration in correlation with neurodegenerative diseases. Adv. Exp. Med. Biol. 854, 119–125. doi: 10.1007/978-3-319-17121-0_17, PMID: 26427402

[ref11] Gorelik E. V. (2014). Pathomorphological examination of hippocampus in the persons of the second period of mature age with cerebral atherosclerosis. J. New Med. Technol. 8, 1–6. doi: 10.12737/5023

[ref12] Guzman-Martinez L. Maccioni R. B. Andrade V. Navarrete L. P. Pastor M. G. Ramos-Escobar N. (2019). Neuroinflammation as a common feature of neurodegenerative disorders. Front. Pharmacol. 10:1008. doi: 10.3389/fphar.2019.01008, PMID: 31572186 PMC6751310

[ref13] Iliff J. J. Wang M. Liao Y. Plogg B. A. Peng W. Gundersen G. A.. (2012). A paravascular pathway facilitates CSF flow through the brain parenchyma and the clearance of interstitial solutes, including amyloid β. Sci. Transl. Med. 4:147ra111. doi: 10.1126/scitranslmed.3003748, PMID: 22896675 PMC3551275

[ref14] Kirabali T. Rust R. Rigotti S. Siccoli A. Nitsch R. M. Kulic L. (2020). Distinct changes in all major components of the neurovascular unit across different neuropathological stages of Alzheimer’s disease. Brain Pathol. 30, 1056–1070. doi: 10.1111/bpa.12895, PMID: 32866303 PMC8018068

[ref15] Kocki J. Ułamek-Kozioł M. Bogucka-Kocka A. Januszewski S. Jabłoński M. Gil-Kulik P.. (2015). Dysregulation of amyloid-β protein precursor, β-secretase, presenilin 1 and 2 genes in the rat selectively vulnerable CA1 subfield of Hippocampus following transient global brain ischemia. J. Alzheimers Dis. 47, 1047–1056. doi: 10.3233/JAD-150299 26401782 PMC4923727

[ref16] Kosicek M. Wunderlich P. Walter J. Hecimovic S. (2014). GGA1 overexpression attenuates amyloidogenic processing of the amyloid precursor protein in Niemann–Pick type C cells. Biochem. Biophys. Res. Commun. 450, 160–165. doi: 10.1016/j.bbrc.2014.05.083 24866237

[ref17] Kress B. T. Iliff J. J. Xia M. Wang M. Wei H. Zeppenfeld D.. (2014). Impairment of paravascular clearance pathways in the aging brain. Ann. Neurol. 76, 845–861. doi: 10.1002/ana.24271, PMID: 25204284 PMC4245362

[ref18] Kudriaeva A. A. Belogurov A. A. (2019). Proteasome: a nanomachinery of creative destruction. Biochemistry 84, 159–192. doi: 10.1134/S0006297919140104, PMID: 31213201

[ref19] Kushnireva L. A. Korkotian E. A. Semyanov A. V. (2019). Undeservedly forgotten: the place of glial cells among the hypothesis of Alzheimer’s disease. Russ. J. Physiol. 105, 1067–1095. doi: 10.1134/S0869813919090085

[ref20] Litvinenko I. V. Emelin A. Y. Lobzin V. Y. Kolmakova K. A. Naumov K. M. Lupanov I. A.. (2019). Amyloid hypothesis of Alzheimer’s disease: past and present, hopes and disappointments. Neurol. Neuropsychiatry Psychosom. 11, 4–10. doi: 10.14412/2074-2711-2019-3-4-10

[ref21] Madsen L. S. Nielsen R. B. Parbo P. Ismail R. Mikkelsen I. K. Gottrup H.. (2022). Capillary function progressively deteriorates in prodromal Alzheimer’s disease: a longitudinal MRI perfusion study. Aging Brain 2:100035. doi: 10.1016/j.nbas.2022.100035, PMID: 36908896 PMC9997144

[ref22] Madsen L. S. Parbo P. Ismail R. Gottrup H. Østergaard L. Brooks D. J.. (2023). Capillary dysfunction correlates with cortical amyloid load in early Alzheimer’s disease. Neurobiol. Aging 123, 1–9. doi: 10.1016/j.neurobiolaging.2022.12.006, PMID: 36610198

[ref23] McGuire P. J. (2019). Mitochondrial dysfunction and the aging immune system. Biology 8:E26. doi: 10.3390/biology8020026, PMID: 31083529 PMC6627503

[ref24] Millien G. Wang H. Zhang Z. Alkon D. L. Hongpaisan J. (2022). PKCε activation restores loss of PKCε, manganese superoxide dismutase, vascular endothelial growth factor, and microvessels in aged and Alzheimer’s disease hippocampus. Front. Aging Neurosci. 14:836634. doi: 10.3389/fnagi.2022.836634, PMID: 35299945 PMC8922019

[ref25] Moeini M. Lu X. Avti P. K. Damseh R. Bélanger S. Picard F.. (2018). Compromised microvascular oxygen delivery increases brain tissue vulnerability with age. Sci. Rep. 8:8219. doi: 10.1038/s41598-018-26543-w, PMID: 29844478 PMC5974237

[ref26] Nagelhus E. A. Ottersen O. P. (2013). Physiological roles of aquaporin-4 in brain. Physiol. Rev. 93, 1543–1562. doi: 10.1152/physrev.00011.2013, PMID: 24137016 PMC3858210

[ref27] Nalivaeva N. N. (2022). The effect of prenatal hypoxia on the metabolism of amyloid precursor protein. Neurochem. J. 16, 219–227. doi: 10.1134/S1819712422030084

[ref28] Nielsen R. B. Egefjord L. Angleys H. Mouridsen K. Gejl M. Møller A.. (2017). Capillary dysfunction is associated with symptom severity and neurodegeneration in Alzheimer’s disease. Alzheimers Dement. 13, 1143–1153. doi: 10.1016/j.jalz.2017.02.007 28343848

[ref29] Nikolenko V. N. Rizaeva N. A. Bulygin K. V. Anokhina V. M. Bolotskaya A. A. (2022). The role of oxidative stress in the development of Alzheimer’s disease. Neurol. Neuropsychiatry Psychosom. 14, 68–74. doi: 10.14412/2074-2711-2022-4-68-74

[ref30] Novikov V. Y. Levchenkova O. S. (2013). Hypoxia-inducible factor as a pharmacological target. Rev. Clin. Pharmacol. Drug Ther. 11, 8–16. doi: 10.17816/RCF1128-16

[ref31] Ong W. Y. Tanaka K. Dawe G. S. Ittner L. M. Farooqui A. A. (2013). Slow excitotoxicity in Alzheimer’s disease. J. Alzheimers Dis. 35, 643–668. doi: 10.3233/JAD-121990 23481689

[ref32] Østergaard L. Aamand R. Gutiérrez-Jiménez E. Ho Y. C. Blicher J. U. Madsen S. M.. (2013). The capillary dysfunction hypothesis of Alzheimer’s disease. Neurobiol. Aging 34, 1018–1031. doi: 10.1016/j.neurobiolaging.2012.09.011 23084084

[ref33] Østergaard L. Jespersen S. N. Engedahl T. Gutiérrez Jiménez E. Ashkanian M. Hansen M. B.. (2015). Capillary dysfunction: its detection and causative role in dementias and stroke. Curr. Neurol. Neurosci. Rep. 15:37. doi: 10.1007/s11910-015-0557-x, PMID: 25956993 PMC4441906

[ref34] Paris D. Patel N. DelleDonne A. Quadros A. Smeed R. Mullan M. (2004). Impaired angiogenesis in a transgenic mouse model of cerebral amyloidosis. Neurosci. Lett. 366, 80–85. doi: 10.1016/j.neulet.2004.05.017, PMID: 15265595

[ref35] Pigolkin Y. I. Zolotenkova G. V. (2014). Age-specific changes in the cerebral cortex capillaries. Sud. Med. Ekspert. 57, 4–6. 25275176

[ref36] Popugaeva E. Pchitskaya E. Bezprozvanny I. (2018). Dysregulation of intracellular calcium signaling in Alzheimer’s disease. Antioxid. Redox Signal. 29, 1176–1188. doi: 10.1089/ars.2018.7506, PMID: 29890840 PMC6157344

[ref37] Riederer B. M. Leuba G. Vernay A. Riederer I. M. (2011). The role of the ubiquitin proteasome system in Alzheimer’s disease. Exp. Biol. Med. 236, 268–276. doi: 10.1258/ebm.2010.010327 21383047

[ref38] Simon M. Wang M. X. Ismail O. Braun M. Schindler A. G. Reemmer J.. (2022). Loss of perivascular aquaporin-4 localization impairs glymphatic exchange and promotes amyloid β plaque formation in mice. Alzheimers Res. Ther. 14:59. doi: 10.1186/s13195-022-00999-5, PMID: 35473943 PMC9040291

[ref39] Soman S. Raghavan S. Rajesh P. G. Varma R. P. Mohanan N. Ramachandran S. S.. (2021). Relationship between cerebral perfusion on arterial spin labeling (ASL) MRI with brain volumetry and cognitive performance in mild cognitive impairment and dementia due to Alzheimer’s disease. Ann. Indian Acad. Neurol. 24, 559–565. doi: 10.4103/aian.AIAN_848_20, PMID: 34728951 PMC8513975

[ref40] Sun X. Bromley-Brits K. Song W. (2012). Regulation of β-site APP-cleaving enzyme 1 gene expression and its role in Alzheimer’s disease. J. Neurochem. 120, 62–70. doi: 10.1111/j.1471-4159.2011.07515.x 22122349

[ref41] Tan J. Evin G. (2012). Β-site APP-cleaving enzyme 1 trafficking and Alzheimer’s disease pathogenesis. J. Neurochem. 120, 869–880. doi: 10.1111/j.1471-4159.2011.07623.x 22171895

[ref42] Tatarnikova O. G. Orlov M. A. Bobkova N. V. (2015). Beta-amyloid and tau-protein: structure, interaction, and prion-like properties. Biochemistry 80, 1800–1819. doi: 10.1134/S000629791513012X, PMID: 26878581

[ref43] Vorobev S. V. Emelin A. Y. Kuzneczova R. N. Kudryavcev I. V. (2020). The role of the immune response in the pathogenesis of Alzheimer’s disease and the possibility of anti-inflammatory therapy. Neurol. Bull. LII, 55–62. doi: 10.17816/nb34654

